# *Methanosarcina acetivorans* contains a functional ISC system for iron-sulfur cluster biogenesis

**DOI:** 10.1186/s12866-020-02014-z

**Published:** 2020-10-23

**Authors:** Thomas M. Deere, Divya Prakash, Faith H. Lessner, Evert C. Duin, Daniel J. Lessner

**Affiliations:** 1 Department of Biological Sciences, University of Arkansas-Fayetteville, Fayetteville, AR 72701 USA; 2 Department of Biochemistry and Molecular Biology, The Pennsylvania State University, University Park, PA 16802 USA; 3 Department of Chemistry and Biochemistry, Auburn University, Auburn, AL 36849 USA

## Abstract

**Background:**

The production of methane by methanogens is dependent on numerous iron-sulfur (Fe-S) cluster proteins; yet, the machinery involved in Fe-S cluster biogenesis in methanogens remains largely unknown. Methanogen genomes encode uncharacterized homologs of the core components of the ISC (IscS and IscU) and SUF (SufBC) Fe-S cluster biogenesis systems found in bacteria and eukaryotes. *Methanosarcina acetivorans* contains three *iscSU* and two *sufCB* gene clusters. Here, we report genetic and biochemical characterization of *M. acetivorans iscSU2*.

**Results:**

Purified IscS2 exhibited pyridoxal 5′- phosphate-dependent release of sulfur from L-cysteine. Incubation of purified IscU2 with IscS2, cysteine, and iron (Fe^2+^) resulted in the formation of [4Fe-4S] clusters in IscU2. IscU2 transferred a [4Fe-4S] cluster to purified *M. acetivorans* apo-aconitase. IscU2 also restored the aconitase activity in air-exposed *M. acetivorans* cell lysate. These biochemical results demonstrate that IscS2 is a cysteine desulfurase and that IscU2 is a Fe-S cluster scaffold. *M. acetivorans* strain DJL60 deleted of *iscSU2* was generated to ascertain the in vivo importance of IscSU2. Strain DJL60 had Fe-S cluster content and growth similar to the parent strain but lower cysteine desulfurase activity. Strain DJL60 also had lower intracellular persulfide content compared to the parent strain when cysteine was an exogenous sulfur source, linking IscSU2 to sulfur metabolism.

**Conclusions:**

This study establishes that *M. acetivorans* contains functional IscS and IscU, the core components of the ISC Fe-S cluster biogenesis system and provides the first evidence that ISC operates in methanogens.

**Supplementary information:**

**Supplementary information** accompanies this paper at 10.1186/s12866-020-02014-z.

## Background

Iron-sulfur (Fe-S) clusters are ubiquitous protein cofactors that are involved in numerous cellular processes, such as respiration, photosynthesis, DNA repair, and regulation. A primary function of Fe-S clusters in proteins is to mediate the transfer of electrons during oxidation-reduction reactions [1]. As such, Fe-S proteins serve critical roles in energy-conservation pathways in almost all organisms and in steps leading to the production of valuable metabolic products, including biofuels (e.g. H_2_) [2, 3]. Simple Fe-S clusters include [2Fe-2S], [3Fe-4S], and [4Fe-4S] clusters, with the [4Fe-4S] cluster being the most prevalent [1, 4]. The metabolism of many organisms also relies on enzymes that use more complex Fe-S clusters, such as those found in the biotechnology relevant enzymes hydrogenase, carbon monoxide dehydrogenase, and nitrogenase [5–7]. For example, nitrogenase contains a [8Fe-7S] cluster and a Mo-8Fe-9S-C-homocitrate cluster, in addition to [4Fe-4S] clusters [8].

Although Fe-S clusters in proteins are typically oxygen-labile, aerobes rely on Fe-S proteins as obligate components of respiratory systems [1]. However, Fe-S proteins are far more abundant in strict anaerobes, specifically those that grow by respiration. Among anaerobes, methanogenic archaea (methanogens) and acetogenic bacteria (acetogens) are predicted to contain the highest number of [4Fe-4S] cluster proteins, indicating that this cluster is critical for methanogenesis and acetogenesis [4, 9]. Methanogenesis is a critical step in the global carbon cycle and in the production of methane as a biofuel. Numerous Fe-S proteins are involved in methanogenesis [10–12]. For example, [4Fe-4S] cluster-containing ferredoxin serves as a primary electron carrier and [4Fe-4S] cluster-containing heterodisulfide reductase plays a central role, including in electron bifurcation [13, 14]. Recently, the bifurcating hydrogenase/heterodisulfide reductase complex was shown to be a dimer of protomers containing 22 [4Fe-4S] clusters, while the bifunctional formyl-methanofuran dehydrogenase complex, which catalyzes the reversible reduction of CO_2_ to formyl-methanofuran, was shown to contain a remarkable 46 electronically coupled [4Fe-4S] clusters [15]. Many additional [4Fe-4S] proteins are involved in the metabolism of methanogens, including several biosynthesis enzymes and regulatory proteins. Several information processing enzymes in methanogens also harbor [4Fe-4S] clusters, such as RNA polymerase [16]. In addition, methanogens, along with related anaerobic methanotrophs, are the only archaea that possess nitrogenase and are therefore capable of nitrogen fixation [17–19]. Despite methanogenesis having an absolute requirement for Fe-S proteins, the factors and mechanisms used by methanogens to assemble and traffic simple and complex Fe-S clusters remain largely unknown.

Two generalized systems (ISC and SUF) are known to function in the biogenesis of Fe-S clusters in bacteria and eukaryotes. A third system (NIF) is specific to biogenesis of the simple and complex Fe-S clusters in the components of nitrogenases found in bacteria [20–23]. For general Fe-S cluster biogenesis, bacteria typically have ISC but may have SUF alone or both ISC and SUF systems (e.g. *Escherichia coli*). ISC is the primary system in *E. coli*, whereas SUF appears important during times of increased oxidative stress and/or Fe limitation [20]. In eukaryotes, the ISC system functions in mitochondria, and the SUF system is primarily present in chloroplasts [23, 24]. The core components of all three systems include a pyridoxal 5′- phosphate (PLP)-dependent cysteine desulfurase (IscS, SufS, or NifS) that liberates sulfur from cysteine, forming a persulfide, followed by sulfur transfer to an Fe-containing scaffold (IscU, SufB(D)C, or NifU). The Fe-S cluster is assembled on the scaffold and subsequently delivered to target apo-proteins, often with the help of accessory and/or carrier proteins. Other accessory proteins may also be involved in cluster assembly [20, 22, 23]. The core scaffold of the SUF system (SufBC) appears universally encoded in the genomes of archaea, and many archaeal genomes also encode homologs of the minimal components of the ISC system (IscS and IscU) [25]. The functional role(s) of these components in archaea are poorly understood.

All sequenced methanogens contain at least one *sufBC* gene cluster, typically arranged as *sufC* then *sufB* [25]. Thus, SufBC may serve as a general Fe-S cluster scaffold in all methanogens. Many sequenced methanogen genomes also encode homologs of IscS and IscU, typically arranged as *iscSU*. Methanogen genomes do not encode NifS or NifU, indicating that methanogens lack a nitrogenase-specific Fe-S cluster biogenesis system. To begin to understand the role and importance of IscSU to methanogens, we report here the genetic and biochemical characterization of IscSU from the genetically tractable methanogen *Methanosarcina acetivorans*.

## Results

### *M. acetivorans* contains three distinct *iscSU* gene clusters

The genome of *M. acetivorans* contains three *isc* gene clusters, each arranged as *iscSU*, and lacking the additional genes found in bacteria, such as in the well-characterized *isc* operon of *E. coli* (Fig. S1) [26]. We have designated the three *iscSU* clusters in *M. acetivorans* as *isc1*, *isc2*, and *isc3*, based on gene annotation order. The *iscS1* and *iscU1* genes are clustered with four additional genes of unknown function. A similar gene arrangement is found in other *Methanosarcina* species including *Methanosarcina barkeri* and *Methanosarcina mazei*. The *iscS2* and *iscU2* genes are clustered with genes encoding enzymes involved in methionine and NAD biosynthesis. A similar gene arrangement is present in many Methanomicrobia, including *M. barkeri.* The genes encoding IscS3 and IscU3 are not clustered with additional genes. A similar gene cluster is found in both *M. barkeri* and *M. mazei*.

IscS1, IscS2, and IscS3 share 45–61% sequence identity to each other, and each is similar in molecular weight and sequence identity to well-characterized IscS from *E. coli* (Table 1). The PLP-binding and active site residues identified in *E. coli* IscS are conserved in the *M. acetivorans* IscS homologs, except for PLP-binding residues in IscS1 (Table 1 and Fig. S2) [27]. IscU1, IscU2, and IscU3 share 49–68% sequence identity to each other, and each protein also has > 50% sequence identity to *E. coli* IscU (Table 1). The Fe-S cluster binding/transfer and Hsp70 chaperone (HscA)-interacting residues (LPPVK) identified in *E. coli* IscU are conserved in the three *M. acetivorans* IscU proteins [24, 28]. However, one of the cysteines involved in Fe-S cluster binding by *E. coli* IscU is replaced with histidine in *M. acetivorans* IscU3 (Table 1 and Fig. S3). Outside of methanogens, *M. acetivorans* IscS and IscU homologs have highest sequence identity (50–68%) to putative IscS and IscU proteins found in Clostridia (e.g. *Ruminiclostridium thermocellum*), consistent with some genes in *Methanosarcina* acquired from *Clostridia* via horizontal transfer [29]. These results indicate that *M. acetivorans* possesses three distinct copies of the core components of the ISC system. However, some of the functionally important residues in *E. coli* IscS and IscU are not conserved in IscS1 and IscU3, respectively, Furthermore, only IscS2 and IscU2 have been consistently detected in the proteome of *M. acetivorans* [30–33], indicating that IscS2 and IscU2 may serve as the primary ISC system. Thus, IscS2 and IscU2 were chosen for initial biochemical and genetic characterization.

**Table 1:** Comparison of predicted *M. acetivorans* IscS and IscU

Canonical Protein (kDa)	Functional motif(s) or residues	*M. acetivorans* homolog	Gene ID:	Percent identity to *E. coli* IscS/U	Motif or residues in corresponding *M. acetivorans* protein
*E. coli* IscS (45.1 kDa)	323-SSGSACTS-330^*a*^	IscS1: MA0808^d^ (42.2 kDa)	MA_RS04215	43	318-STGSACFS-325
		IscS2: MA2718^d^ (43.3 kDa)	MA_RS14225	48	232-STGSACNS-330
		IscS3: MA3264 (43.6 kDa)	MA_RS17030	48	317-STGSACSS-324
*E. coli* IscU (13.8 kDa)	C37, D39, C63, C106^b^ 99-LPPVK-103^*c*^	IscU1: MA0807 (24.2 kDa)	MA_RS04210	50	C45, D47, C72, C116 109-LPPIK-113
		IscU2: MA2717^d^ (13.9 kDa)	MA_RS14220	54	C34, D36, C59, C103 96-LPPIK-100
		IscU3: MA3265 (14.0 kDa)	MA_RS17035	50	H32, D34, C57, C101 94-LPPGK-98

^a^ Cysteine desulfurase active site motif

^b^ Residues critical for Fe-S cluster binding and transfer

^c^ Residues critical for interaction with the HscA chaperone

^d^ Detected in *M. acetivorans* proteome (see text)

### IscS2 is a cysteine desulfurase

**Fig. 1:**
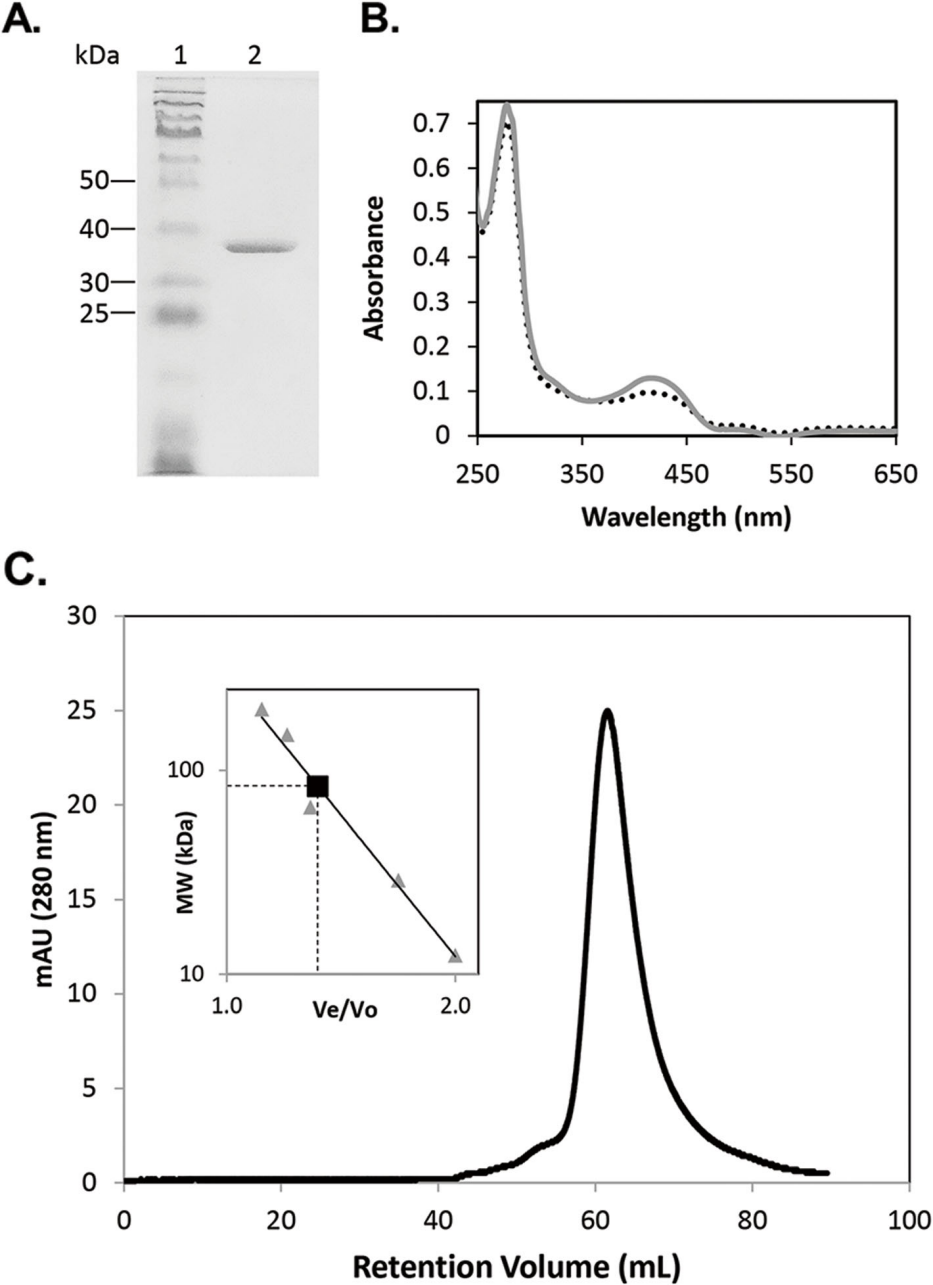
Purified IscS2 binds PLP and is a homodimer. **a** SDS-PAGE analysis of purified IscS2 (cropped image of original). Lane 1, MW marker; lane 2, IscS2 (2.5 μg). **b** UV-visible spectra of 20 μM IscS2 (dotted line) or IscS2^PLP^ (solid line) in 50 mM Tris pH 7.2, 150 mM NaCl. **c** Size-exclusion chromatography of IscS2. IscS2 (3.3 mg loaded) was analyzed by size-exclusion chromatography with 50 mM Tris pH 8.0, 150 mM NaCl, 2 mM DTT, 10% glycerol. The molecular weight of IscS2 was calculated from a standard curve (inset). The square represents the V_e_/V_o_ of IscS2 with a calculated molecular weight of 84 kDa, consistent with homodimer (87 kDa)

Recombinant IscS2 was over-produced in *E. coli* and purified to homogeneity (Fig. 1a). Purified IscS2 was pale-yellow and exhibited an UV-visible spectrum with an absorbance maximum at 420 nm (Fig. 1b), consistent with the presence of PLP [34]. Purified IscS2 was able to remove sulfur from L-cysteine (Table 2) confirming IscS2 is a cysteine desulfurase. To determine if the cysteine desulfurase activity of IscS2 is dependent on PLP and if recombinant IscS2 contained full incorporation of PLP, assays were performed in the presence and absence of PLP (Table 2). A 57% increase in the cysteine desulfurase activity of IscS2 was observed when PLP was added to the assay. IscS2 reconstituted with PLP (IscS2^PLP^) exhibited the same specific activity both in the absence and presence of additional PLP. An increase in the absorbance at 420 nm was also observed with IscS2^PLP^ (Fig. 1b). These data are consistent with full incorporation of PLP in IscS2^PLP^. IscS2^PLP^ was used in all subsequent experiments. Size-exclusion chromatography of IscS2^PLP^ revealed the purified protein exists as a homodimer (Fig. 1c), similar to previously characterized IscS [27, 34]. Taken together, these results reveal that IscS2 is a PLP-dependent cysteine desulfurase.

**Table 2:** Effect of PLP on cysteine desulfurase activity of purified IscS2

Sample	Cysteine desulfurase activity^*b*^
IscS2	21.5 ± 1.2
IscS2 + PLP	33.8 ± 0.5
IscS2^PLP*a*^	35.1 ± 2.1
IscS2^PLP^ + PLP	33.4 ± 0.5

^a^ IscS2 reconstituted with PLP

^*b*^ Cysteine desulfurase activity (nmol sulfur min^− 1^ mg^− 1^ IscS2) of 5 μM IscS2 or IscS2^PLP^ in the absence or presence of additional PLP (50 μM). Results are means from triplicates ±1 standard deviation

### IscU2 is capable of binding Fe-S clusters

**Fig. 2:**
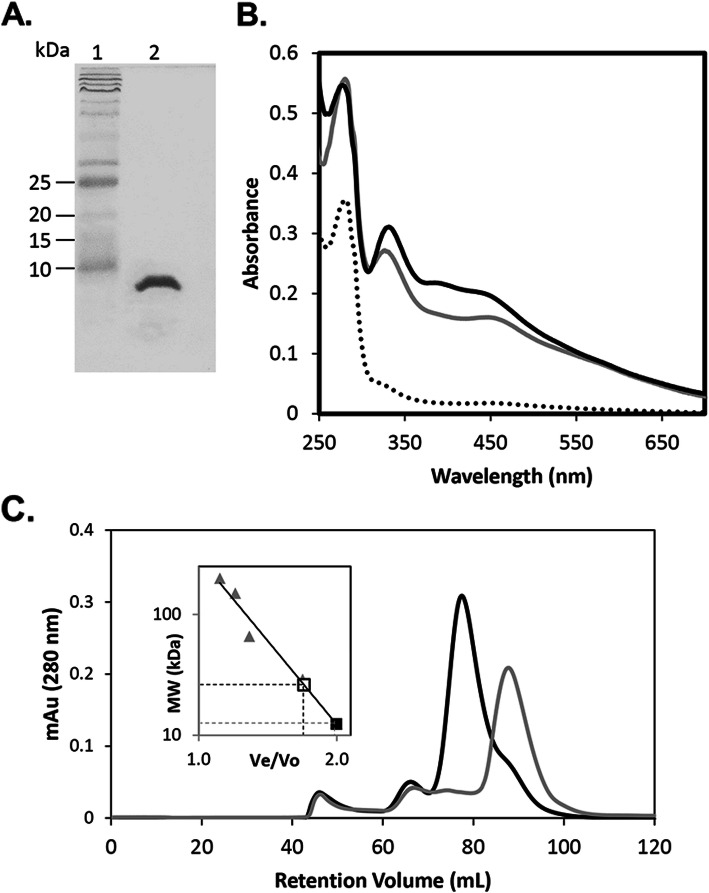
Fe-S cluster reconstitution of purified IscU2. **a** SDS-PAGE analysis of purified IscU2 (cropped image of original). Lane 1, MW marker; lane 2, IscU2 (2.4 μg). **b** Anaerobic UV-visible spectra of 20 μM IscU2 (dotted line), IscU2^S-FeS^ (black line) and IscU2^C-FeS^ (gray line) in 50 mM Tris pH 7.2, 150 mM NaCl. **c** Anaerobic size exclusion chromatography of 14.8 mg of IscU2 (gray line) and 15.6 mg of IscU2^C-FeS^ (black line) in 50 mM Tris pH 8.0, 150 mM NaCl, 2 mM DTT, 10% glycerol. The molecular weight of IscU2 and IscU2^C-FeS^ were calculated with a standard curve (inset). The calculated MW of IscU2 (solid square symbol) was 12 kDa and the calculated MW of IscU2^C-FeS^ (open square symbol) was 26 kDa

Recombinant IscU2 was expressed in *E. coli* and purified to homogeneity under anoxic conditions (Fig. 2a). Purified IscU2 was pale-red and exhibited an UV-visible spectrum with minor absorbance maxima at 360 nm and 438 nm (Fig. 2b). As-purified IscU2 contained both iron and acid-labile sulfide (Table 3). However, the A_438_/A_280_ ratio and the iron/sulfide content was low indicating that a substantial portion of purified IscU2 was devoid of Fe-S clusters. IscU devoid of cluster (apo-IscU) typically exists as a monomer and subsequently dimerizes upon incorporation of [2Fe-2S] clusters. The two [2Fe-2S] clusters in dimeric IscU can then reductively couple to form a single [4Fe-4S] cluster [35, 36]. Size-exclusion chromatography of as-purified IscU2 yielded a major peak consistent with monomeric IscU2 and a minor peak consistent with dimeric IscU2 (Fig. 2c), indicating that the majority of as-purified IscU2 is in the apo-form. To test the ability of IscU2 to bind Fe-S clusters, IscS2-dependent and chemical-dependent reconstitution of Fe-S clusters in IscU2 were performed. IscS2-dependent reconstituted IscU2 (IscU2^S-FeS^) was generated by the anoxic incubation of as-purified IscU2 with cysteine, iron and a catalytic amount of IscS2. Chemical-dependent reconstituted IscU (IscU2^C-FeS^) was generated by the anoxic incubation of as-purified IscU2 with a molar excess of sodium sulfide and iron. A substantial increase in the iron and acid-labile sulfur content and the A_438_/A_280_ ratio was observed for both IscU2^S-FeS^ and IscU2^C-FeS^ (Table 3), consistent with an increase in Fe-S clusters in both samples. The UV-visible spectra of IscU2^S-FeS^ and IscU2^C-FeS^ were similar and showed a substantial increase in the absorbance at 360 nm and 438 nm (Fig. 2b). Size-exclusion chromatography revealed IscU2^C-FeS^ was dimeric (Fig. 2c), demonstrating that Fe-S cluster binding causes dimerization of IscU2. The UV-visible spectra and levels of iron and sulfur in IscU2^S-FeS^ and IscU2^C-FeS^ are consistent with the presence of a two [2Fe-2S] clusters or a single [4Fe-4S] cluster per dimer.

**Table 3:** Comparison of the properties of purified *M. acetivorans* IscU2

Protein	A_438_/A_280_	ε_438_ (mM^−1^ cm^− 1^)	Iron^a^	Sulfide^b^
IscU2	0.10	0.90	0.57 ± 0.06	0.44 ± 0.04
IscU2^C-FeS^	0.26	6.07	2.47 ± 0.11	1.99 ± 0.24
IscU2^S-FeS^	0.27	5.91	3.15 ± 0.24	3.15 ± 0.39

^a^ nmol iron/nmol of IscU2

^b^ nmol acid-labile sulfide/nmol of IscU2

**Fig. 3:**
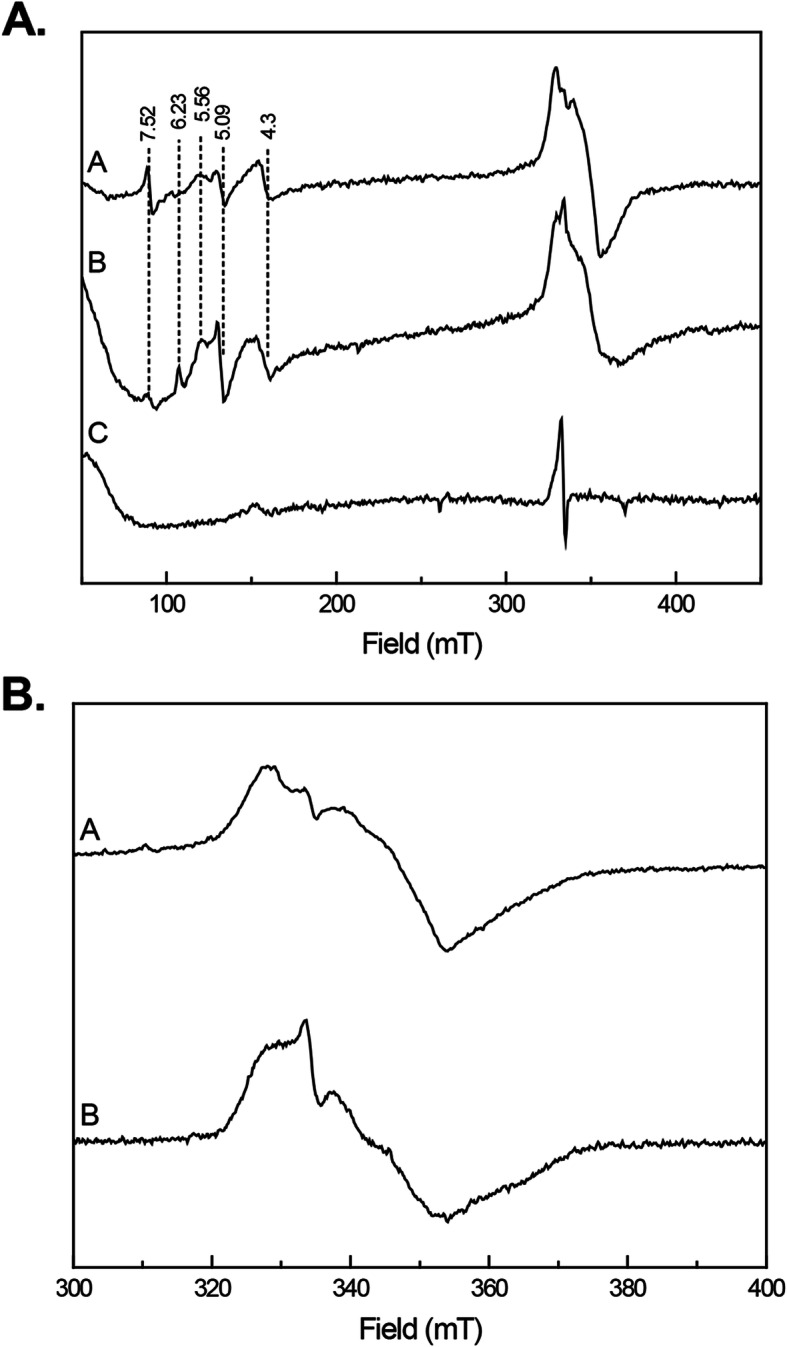
EPR spectra of IscU2. **a** Expanded view, trace A: IscU2^C-FeS^ (100 μM) reduced with dithionite, trace B: IscU2^S-FeS^ (100 μM) reduced with dithionite, trace C: IscU2^S-FeS^ (100 μM) as such. Numbers shown represent *g* values. **b** Detailed view, trace A: IscU2^C-FeS^ reduced with dithionite, trace B: IscU2^S-FeS^ reduced with dithionite

EPR analysis of the as-purified IscU2 did not produce a signal under the conditions tested (data not shown), likely due to the low Fe-S cluster content. However, both IscU2^S-FeS^ and IscU2^C-FeS^ generated EPR spectra upon the addition of dithionite (Fig. 3). Both reduced samples showed a similar EPR signal that can be attributed to [4Fe-4S]^+^ clusters, consistent with reductive coupling of the [2Fe-2S] clusters into a single [4Fe-4S] in dimeric IscU2 [35]. From the presence of signals at around *g* = 2 (330 mT) and higher *g* values (lower field values) it can be concluded that the clusters display several different spin states. The signal in the 300–400 mT region are due to S = 1/2 species. The peak at *g* = 4.3 is due to the spin ±3/2 doublet of an S = 5/2 species with E/D = 0.333. This peak can be due to a 4Fe cluster or adventitiously bound iron. The peaks at *g* = 5.09 and 6.23 are due to the spin ±1/2 doublet of an S = 5/2 species with E/D = 0.032, the peak at *g* = 5.56 is due to the same spin state but is due to the spin ±3/2 doublet. The peak at *g* = 7.52 is due to the spin ±1/2 doublet of a S = 7/2 species with E/D = 0. The as-such sample (Fig. 3a, trace C) does not show signals due to [4Fe-4S]^2+^ since in that redox state the spin is 0. The sharp signal at around 330 mT is due to a [3Fe-4S]^1+^ species. The multitude of spin states point towards a highly variable environment for the clusters present in the binding site on IscU2. These results are consistent with IscU2 as an Fe-S cluster scaffold protein, whereby IscS2 can catalyze the formation of [4Fe-4S] clusters in apo-IscU2 in the presence of iron and cysteine.

### *M. acetivorans* contains a [4Fe-4S] cluster aconitase

Aconitase is a member of the dehydratase family of enzymes that requires a [4Fe-4S] cluster for activity and is the most common acceptor protein used in Fe-S cluster transfer assays [20]. The activity of aconitase can be measured spectrophotometrically in a coupled assay with isocitrate dehydrogenase, whereby the reduction of NADP^+^ with isocitrate is measured at 340 nm [37]. IscU from several organisms is documented to transfer clusters to apo-aconitase [38–40]. Given the substantial information on cluster transfer to aconitase by IscU, aconitase is an ideal acceptor protein to initially assess cluster transfer from IscU2. *M. acetivorans* encodes a single aconitase homolog (MA0250), and *M. acetivorans* cell lysate contains detectable aconitase activity (5.2 nmol NADPH min^− 1^ mg^− 1^ protein) as measured by the coupled assay. Recombinant MA0250 was expressed in *E. coli* and purified to homogeneity (Fig. S4A). As-purified MA0250 lacked aconitase activity. However, after chemical reconstitution with iron and sulfide, purified MA0250 exhibited robust aconitase activity (100 nmol NADPH min^− 1^ mg^− 1^ protein) and a broad absorbance maximum around 400 nm in UV-visible spectrum, consistent with the presence of a [4Fe-4S] cluster (Fig. S4B). These results reveal that MA0250 is an aconitase (designated here as AcnA) with activity dependent on the presence of a [4Fe-4S] cluster.

### Cluster-loaded IscU2 can restore the activity of *M. acetivorans* apo-aconitase

**Fig. 4:**
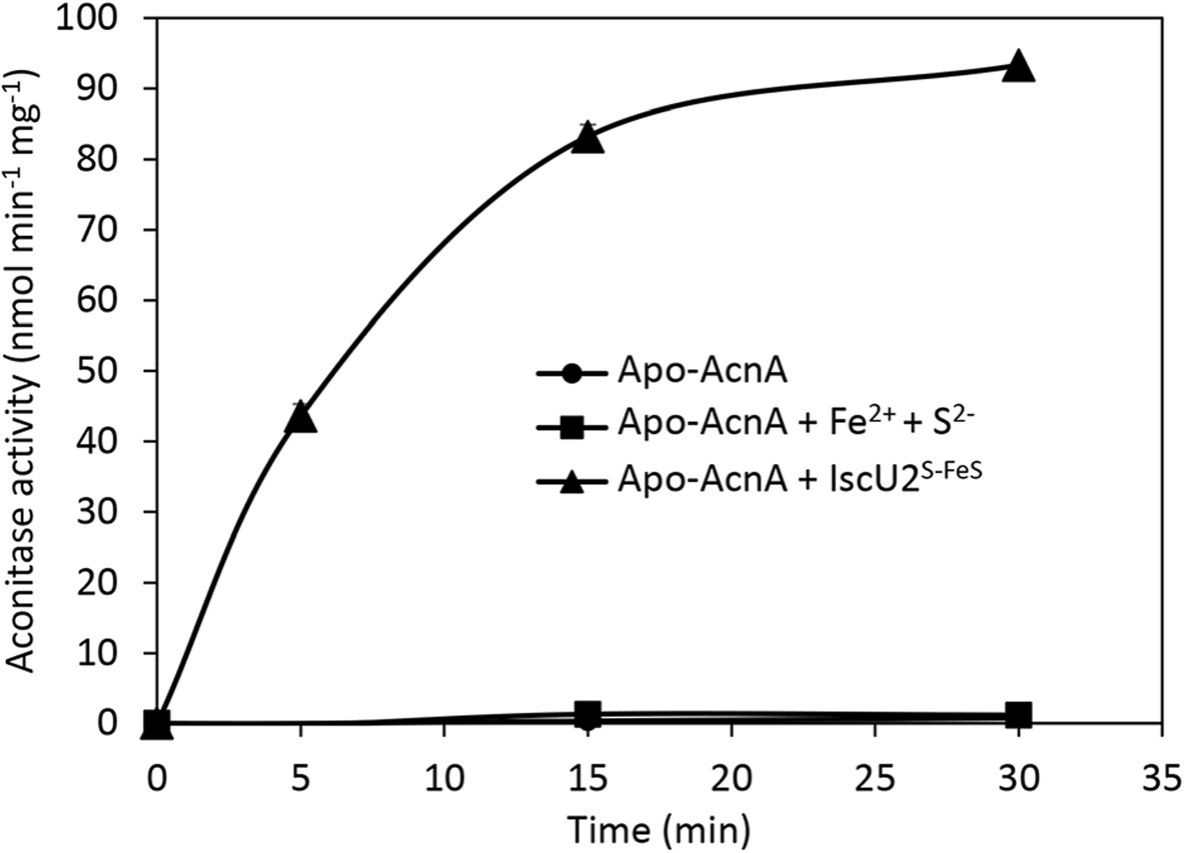
Reconstitution of apo-AcnA activity by [4Fe-4S]-IscU2. Apo-AcnA (4 μM) was incubated with IscU2^S-FeS^ (40 μM) or iron (Fe^2+^) and sulfide (S^2−^) (80 μM each) and aconitase activity was measured over time

Anoxic incubation of apo-AcnA with IscU2^S-FeS^ resulted in rapid and complete recovery of aconitase activity, whereas incubation with iron and sulfide, at the same molar concentration as found in IscU2^S-FeS^, did not restore any activity to apo-AcnA over the same timeframe (Fig. 4). The ability of cluster loaded IscU2 to restore aconitase activity was also examined using cell lysate. Consistent with AcnA containing an oxygen-labile [4Fe-4S] cluster required for activity, exposure of cell lysate to air resulted in a complete loss of aconitase activity, even when the cell lysate was made anoxic again (Table 4). The addition of as-purified IscU2 during the anoxic incubation of air-exposed lysate also did not restore aconitase activity. However, anoxic incubation of air-exposed cell lysate with IscU2^S-FeS^ partially restored aconitase activity (Table 4). Overall, these data demonstrate that cluster-loaded IscU2 is capable of transferring Fe-S clusters to apo-AcnA, consistent with IscU2 as an Fe-S cluster scaffold.

**Table 4:**
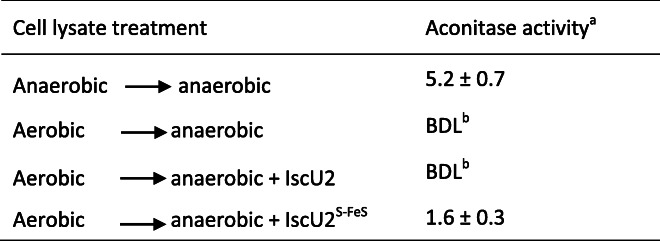
IscU2-dependent recovery of aconitase activity in air-exposed *M. acetivorans* cell lysates

^a^ nmol NADPH min^−1^ mg^− 1^ protein; Results are means from triplicates ±1 standard deviation

^b^ Below Detection Limit (≥ 0.1 nmol NADPH min^− 1^ mg^− 1^ protein)

### Deletion of *iscSU2* impacts sulfur metabolism in *M. acetivorans*

**Fig. 5:**
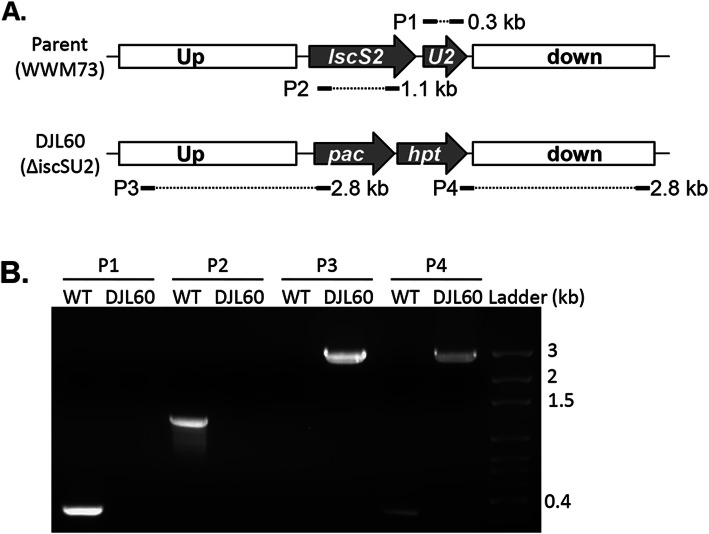
PCR confirmation of *iscSU2* deletion in *M. acetivorans* strain DJL60. **a** Schematic showing replacement of *iscSU2* with *pac-hpt* in strain DJL60 and predicted PCR products are indicated by P1–4. **b** Gel image of products of PCR reactions P1–4 with WWM73 and DJL60 genomic DNA

The results from the biochemical characterization of recombinant IscS2 and IscU2 reveal properties consistent with each protein functioning in Fe-S cluster biogenesis. To determine the importance of IscS2 and IscU2 to *M. acetivorans* physiology, a mutant strain (DJL60) was generated with *iscSU2* deleted and replaced with the *pac-hpt* genes (Fig. 5a). The mutant was isolated using HS medium supplemented with both cysteine and sulfide. PCR (Fig. 5b) and DNA sequencing verified the DJL60 mutant. Thus, neither IscS2 nor IscU2 are essential to *M. acetivorans*.

**Fig. 6:**
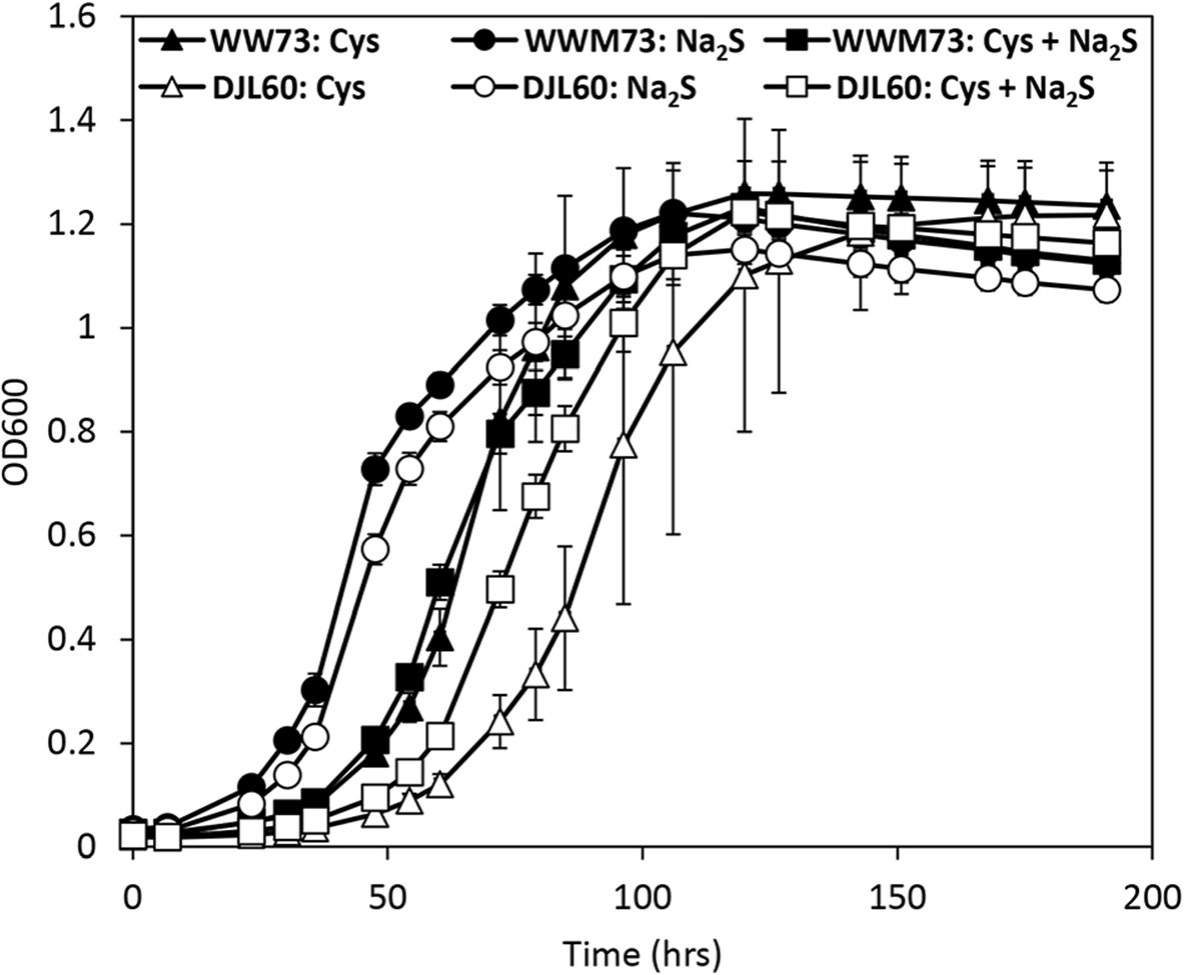
Growth of *M. acetivorans* strains WWM73 and DJL60 with different sulfur sources. Each strain was grown in HS_DTT_ medium containing 125 mM methanol supplemented with 3 mM cysteine (Cys) and/or 3 mM sodium sulfide (Na_2_S). Growth was monitored by the optical density at 600 nm (OD_600_). Data points are the mean of *n* = 3 with error bars ± STD.

To test the impact of the loss of IscSU2 on *M. acetivorans*, first the growth of strain DJL60 with cysteine, sulfide, or cysteine + sulfide was compared to the parent strain WWM73. Growth studies were performed in HS medium supplemented with 1.5 mM dithiothreitol (DTT), designated here as HS_DTT_ medium, to maintain similar redox conditions with the different sulfur sources. DTT cannot be used as a sulfur source by *M. acetivorans* [41]. The growth profiles of strains WWM73 and DJL60 were similar with the different exogenous sulfur sources (Fig. 6). However, compared to strain WWM73, strain DJL60 exhibited slightly slower and more variable growth when cysteine was present, especially when cysteine was the only sulfur source.

**Fig. 7:**
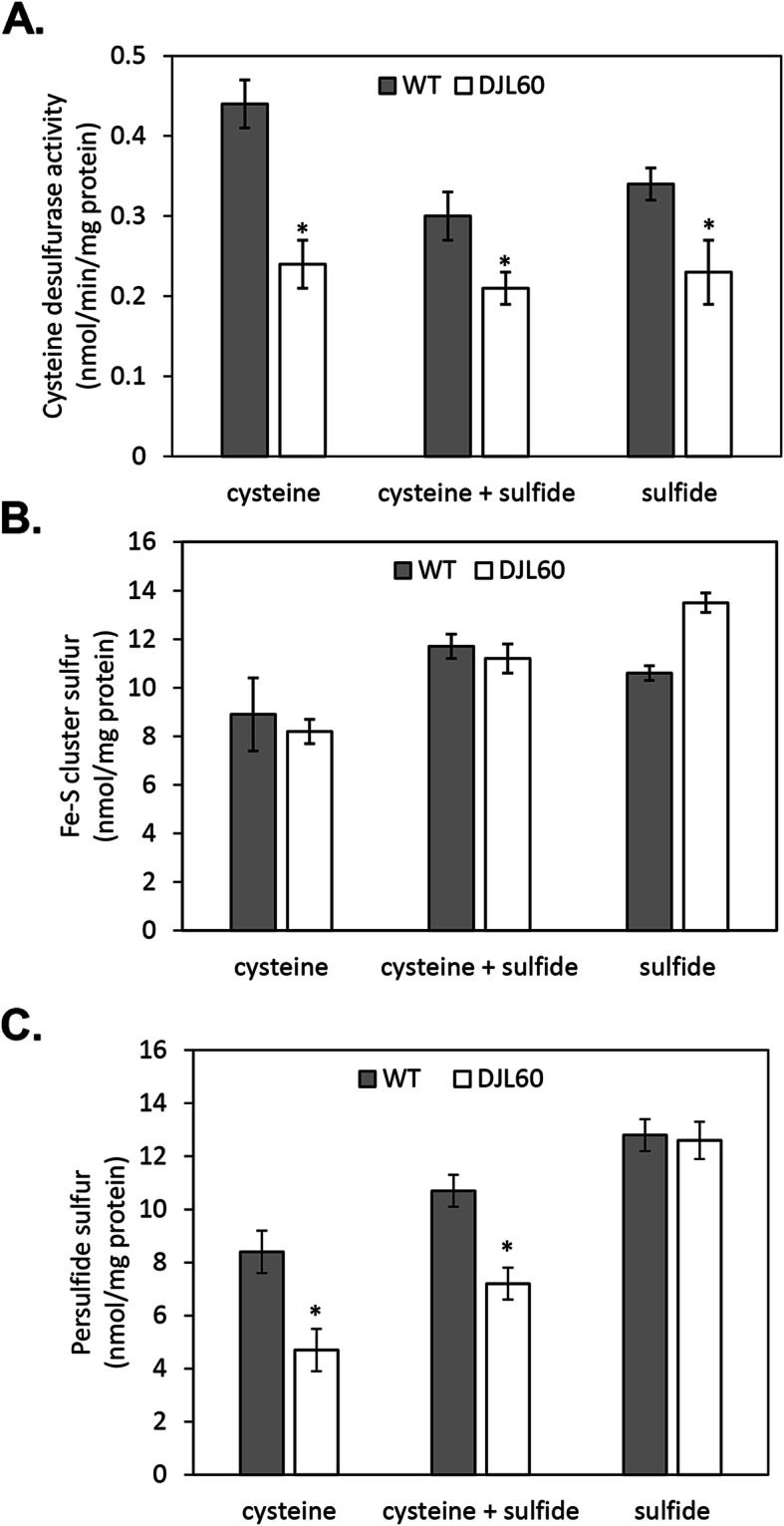
Cysteine desulfurase activity, Fe-S cluster, and persulfide levels in cell lysates of strains WWM73 and DJL60. Cysteine desulfurase activity (**a**), Fe-S cluster content (**b**) and persulfide content (**c**) were measured as described in methods. Asterisks indicate significant difference between WWM73 and DJL60 lysate, as determined by t-test (*p* < 0.02 for A, *p* < 0.01 for C)

To determine the impact of the loss of IscSU2 on sulfur metabolism of *M. acetivorans* with the different exogenous sulfur sources, cysteine desulfurase activity, Fe-S cluster levels and persulfide content in lysate from strain DJL60 and WWM73 cells were determined. Importantly, lysate from strain DJL60 grown under all conditions exhibited significantly less cysteine desulfurase activity than strain WWM73 lysate (Fig. 7a), consistent with IscS2 as a functional in vivo cysteine desulfurase. However, cysteine desulfurase activity was not completely abolished in strain DJL60 indicating additional enzymes (e.g. IscS3) contribute to the total cysteine desulfurase activity. Interestingly, lysate from *Methanococcus maripaludis*, whose genome does not encode a cysteine desulfurase, contains some cysteine desulfurase activity from an unknown source [42]. No significant difference was observed in the Fe-S cluster content in strain DJL60 and WWM73 lysate across all sulfur conditions (Fig. 7b). However, the persulfide content in strain DJL60 was significantly lower in lysate from cysteine and cysteine + sulfide grown cells compared to lysate from WWM73 cells (Fig. 7c). Overall, these results confirm IscS2 as an in vivo cysteine desulfurase and link IscSU2 to sulfur metabolism in *M. acetivorans*.

## Discussion

Methanogens are metabolic specialists; all species are dependent on methanogenesis for growth [12]. Methanogenesis has an obligate requirement for Fe-S cluster proteins. An understanding of the protein machinery used by methanogens for the de novo synthesis of Fe-S clusters may lead to improved methods to increase or inhibit methanogenesis. The results presented here reveal that *M. acetivorans* harbors functional IscS and IscU, the minimal components of the ISC-type Fe-S cluster biogenesis system, that serve as the general system in numerous bacteria and in mitochondria. It is reasonable to conclude that other methanogens whose sequenced genomes encode IscSU also utilize a minimal ISC system for Fe-S cluster biogenesis.

The absence of IscSU in some methanogens is likely due to physiological differences resulting from environmental constraints. Members of the Methanococcales, Methanopyrales, and some species of Methanobacteriales lack *iscSU* [25, 43]. These species appear to lack any cysteine desulfurase, suggesting that cysteine may not serve as the direct sulfur donor for Fe-S cluster biogenesis. Indeed, experimental evidence revealed that *M. maripaludis* uses sulfide, instead of cysteine, as the sulfur donor for Fe-S cluster biogenesis [42]. This was the first evidence of a substrate, other than cysteine, serving as the sulfur donor for Fe-S cluster biogenesis in any organism. *Methanococcus* spp. live in sulfide-rich environments and are dependent primarily on sulfide as an exogenous source of sulfur and do not use cysteine [44]. Like all methanogens, *M. maripaludis* encodes SufBC. It seems likely that methanogen SufBC does not partner with a cysteine desulfurase but receives sulfur from sulfide for the assembly of an Fe-S cluster. The mechanisms and factors involved in directing sulfide to Fe-S cluster biogenesis machinery are unknown in *Methanococcus*, but presumably involve an unknown protein factor(s) to traffic sulfur from sulfide to SufBC.

Most species of the Methanomicrobia contain at least one copy of *iscSU*, in addition to *sufCB* [25]. All *Methanosarcina* spp. possess *iscSU* [43]. *Methanosarcina* spp. are the most metabolically diverse methanogens capable of producing methane with H_2_/CO_2_, methylated compounds, and acetate. *Methanosarcina* are one of only two genera capable of metabolizing acetate, which accounts for two-thirds of all biogenic methane produced [12]. *Methanosarcina* also have the largest genomes and are the most oxygen tolerant methanogens [26, 45]. Unlike *Methanococci*, *Methanosarcina* can use cysteine, in addition to sulfide, as an exogenous sulfur source. The acquisition of IscSU, in addition to SufBC, may aid in the metabolic diversity and aerotolerance of *Methanosarcina* by conferring the ability to use cysteine as an exogenous sulfur source and as a direct sulfur source for the biogenesis of Fe-S clusters in more oxidizing environments with limited sulfide.

*M. acetivorans* and related Methanosarcinales contain multiple copies of *iscSU*. *M. acetivorans* IscSU1–3 may be functionally redundant, which is supported by residual cysteine desulfurase activity and normal Fe-S cluster content in strain DJL60. However, IscS1 and IscU3 lack some of the residues required for the function of *E. coli* IscS and IscU, indicating these orthologs could be non-functional. Alternatively, each ortholog may serve a different function in Fe-S cluster biogenesis. Recently, the structure of a recombinant [2Fe-2S] cluster-containing IscS-IscU complex from *Archaeoglobus fulgidus* was solved [46]. *A. fulgidus* is an anaerobic archaeon that is closely related to methanogens. Interestingly, *A. fulgidus* IscS lacks cysteine desulfurase activity due to a substitution of a catalytically essential lysine with aspartate. In the solved structure, *A. fulgidus* IscS provides a cysteine ligand to the [2Fe-2S] cluster in IscU, suggesting it plays a role in cluster assembly as a ligand, but not by providing sulfur [46–48]. Like *A. fulgidus* IscS, IscS1 lacks the catalytically essential lysine (Fig. S2) indicating it may function as *A. fulgidus* IscS. Expression of recombinant IscS1 in *E. coli* led to the formation of inclusion bodies, indicating it may require co-expression with IscU1 for stabilization (data not shown).

Aerobic bacteria and eukaryotes typically have complex ISC and SUF systems that involve several accessory factors, whereas methanogens appear to use only the minimal components, IscSU and SufBC, respectively. It was proposed that the SUF system increased in complexity as additional factors were needed to control iron and sulfur trafficking to synthesize Fe-S clusters in cells that live in more oxidizing environments [25]. The same may be true for the ISC system. For example, the *M. acetivorans isc* gene clusters lack any of the additional genes found in the *E. coli isc* operon, including *hscA* and *hscB*, which are essential to ISC-dependent Fe-S cluster biogenesis in *E. coli*. HscA and HscB are chaperones that specifically interact with IscU to accelerate Fe-S cluster transfer to target apo-proteins. HscA specifically binds to the IscU LPPVK motif to elicit conformational changes in IscU dependent on the hydrolysis of ATP [28, 49]. Surprisingly, *M. acetivorans* IscSU1–3 all contain a variant of the LPPVK motif (Table 1), yet the genome of *M. acetivorans* does not encode homologs of HscA or HscB. It is possible that unrelated chaperones fulfill the role of HscA and HscB. However, it was recently shown that several point mutations in IscU suppress the essential role of HscA and HscB in *E. coli* [50]. Given that *M. acetivorans* cluster-loaded IscU2 rapidly restored the in vitro activity of apo-aconitase in the absence of additional factors, it seems more likely that chaperones are not involved in ISC-dependent Fe-S cluster biogenesis in *M. acetivorans*, despite the presence of the LPPVK motif in IscU1–3.

Finally, the results indicate a cysteine-specific function for *M. acetivorans* IscSU2. Deletion of *iscSU2* resulted in decreased in vivo cysteine desulfurase activity. Although a decrease in Fe-S cluster content was not observed in strain DJL60 grown with cysteine, it is possible that IscU1 and/or IscU3 serve as Fe-S cluster scaffolds in the absence of IscU2. Cysteine desulfurase serves as the central hub for trafficking sulfur in bacteria and eukaryotes. Importantly, a decrease in the persulfide content of DJL60 cells compared to wild type cells was only observed when cysteine was provided as an exogenous sulfur source, indicating IscS2 participates in trafficking sulfur from cysteine, but is not involved when sulfide is the sole sulfur source. Based on results presented here and with *M. maripaludis* [42, 51], cells of *M. acetivorans* may primarily use IscSU for Fe-S cluster biogenesis and sulfur trafficking when provided cysteine, and primarily use SufBC for Fe-S cluster biogenesis when cells are provided sulfide.

## Conclusions

This study provides the first experimental evidence that methanogens possess functional components of the ISC system for Fe-S cluster biogenesis. Biochemical analyses demonstrated that *M. acetivorans* IscS is a cysteine desulfurase and that IscU2 is an Fe-S cluster scaffold. Importantly, IscSU2 can provide Fe-S clusters to target apo-proteins. Deletion of *iscSU2* revealed that IscSU2 is not essential to Fe-S cluster biogenesis in *M. acetivorans*. However, loss of IscSU2 impacts sulfur metabolism. These results provide new insight into the mechanisms of Fe-S cluster biogenesis in methanogens, which may aid in the development of methods to enhance or inhibit methanogenesis, due to the obligate requirement for Fe-S proteins.

## Methods

### M. acetivorans growth

*M. acetivorans* strain WWM73 was obtained from Dr. Bill Metcalf [52] and was used as the parent strain for all experiments. All strains of *M. acetivorans* (Table S1) were grown in HS medium containing 125 mM methanol as a carbon and energy source as previously described [53, 54]. Each liter of HS medium contains 23.4 g NaCl, 3.8 g NaHCO_3_, 1.0 g KCl, 11.0 g MgCl_2_*6H_2_O, 0.3 g CaCl_2_*2H_2_O, 1.0 g NH_4_Cl, 0.5 g L-cysteine, 5 mL of 1 M KH_2_PO_4_ at pH = 7.4, 1 mL of 0.1% w/v resazurin, 10 mL of Wolfe’s Mineral Solution (supplemented with 0.024 g/L NiCl_2_*H_2_O in the stock), and 2 mL of a 5x concentrated Wolfe’s Vitamin Solution [55]. HS medium was made anoxic and dispensed into Balch tubes within an anaerobic chamber (Coy Laboratories) containing 75% N_2_, 20% CO_2_, and 5% H_2_. Standard culture conditions include 0.025% w/v Na_2_S*9H_2_O added from a sterile, anoxic stock just prior to inoculation. To examine growth with different sulfur sources, 1.5 mM DTT was added to HS medium (HS_DTT_ medium) in lieu of cysteine prior to autoclaving, and 3 mM L-cysteine and/or 1 mM sodium sulfide were added from sterile anoxic stock solutions prior to inoculation. Growth was monitored by measuring the optical density at 600 nm (OD_600_) of the culture tubes using a spectrophotometer (Thermo Fisher, Genesys 10 Bio).

### Expression of recombinant *M. acetivorans* IscS2, IscU2, and aconitase in *E. coli*

The genes *iscS2* (MA2718) and *iscU2* (MA2717) were amplified by PCR from *Methanosarcina acetivorans* genomic DNA isolated from wild-type strain C2A using standard guanidine thiocyanate lysis, protein precipitation, and isopropanol extraction. *Nde*I and *Xho*I recognition sites were added at the 5′ and 3′ ends of the PCR product, respectively. The putative *acnA* aconitase gene (MA0250) was similarly amplified, but with an *Nhe*I site rather than *Nde*I. All PCR products were generated using Phusion HF polymerase (New England Biolabs) with reagent concentrations per the manufacturer’s instructions. Annealing temperatures of 60 °C for *iscS2* and *iscU2*, and 67 °C for *acnA*, were used (primers listed in Table S1). The PCR products were digested with *Nde*I or *Nhe*I and *Xho*I and ligated using T4 DNA ligase (New England Biolabs) with pET28a that had been similarly digested, resulting in each gene fused to a thrombin-cleavable His_6_-tag. All enzymes were from New England Biolabs and reactions were carried out per manufacturer’s instructions. Sub-cloning efficiency *E. coli* DH5α competent cells (Invitrogen) were transformed with the ligation reactions and cells harboring plasmids with *iscU2*, *iscS2*, and *acnA* were identified by restriction digests and confirmed by DNA sequencing (Eurofins). For expression of each N-terminal His_6_-tagged recombinant protein, *E. coli* Rosetta (DE3) pLacI was separately transformed with the plasmids containing *iscU2* (pDL201), *iscS2* (pDL202), and *acnA* (pDL204).

*E. coli* Rosetta (DE3) pLacI harboring pDL201, pDL202, or pDL204 were grown in LB medium containing 50 μg/mL kanamycin and 17 μg/mL chloramphenicol with shaking at 37 °C. At an optical density at 600 nm (OD_600_) of 0.6, 0.5 mM IPTG was added, and the temperature was lowered to 25 °C for the IscS2-expression culture or 16 °C for the IscU2- and AcnA-expression cultures. The AcnA-expression culture was also supplemented with 0.5 M D-sorbitol at induction to inhibit inclusion body formation. After 18 h, cells were harvested by centrifugation and frozen at − 80 °C.

### Purification of recombinant proteins

For purification of IscS2, thawed cells were resuspended in buffer A (20 mM Tris pH 8.0, 500 mM NaCl, 10% glycerol) containing a few crystals of DNase I and approximately 1 mM benzamidine HCl hydrate. Cells were lysed by two passages through a French pressure cell at > 110 MPa. Lysates were centrifuged at 41,000 x *g* and 4 °C for 35 min. The supernatant was passed through a 0.45 μm filter and loaded on a chromatography column containing 5 mL of Ni^2+^-agarose resin (Genscript) pre-equilibrated with 25 mL of buffer A. The column was sequentially washed with 50 mL buffer A, 25 mL buffer A containing 10 mM imidazole, and 25 mL buffer A. The column was then incubated in 5 mL buffer A containing 50 U of thrombin (Promega) at 25 °C for 16 h. Protein was eluted from the column by the addition of 10 mL of buffer A followed by 10 mL buffer A containing 250 mM imidazole. Thrombin was removed using a 1 mL HiPrep benzamidine column (GE Healthcare) following the manufacturer’s instructions. Purified IscS2 was exchanged into storage buffer (50 mM Tris pH 8.0, 150 mM NaCl, 10% glycerol) using a PD-10 column (GE Healthcare) and stored at − 80 °C until use.

For purification of IscU2, all steps were performed anaerobically under an atmosphere of 95% N_2_, 5% H_2_ in an anaerobic chamber (Coy Laboratories). Thawed cells were resuspended in buffer B (20 mM Tris pH 8.0, 2 M NaCl, 10% glycerol) containing a few crystals of DNase I and benzamidine HCl hydrate. Cells were lysed by two passages through a French pressure cell at > 110 MPa. Lysates were centrifuged at 41,000 x *g* and 4 °C for 35 min. The supernatant was passed through a 0.45 μm filter and loaded on a 5 mL Ni^2+^-agarose resin chromatography column pre-equilibrated with 25 mL of buffer B. The column was sequentially washed with 50 mL buffer B, 25 mL buffer B containing 10 mM imidazole, 25 mL buffer B, and 25 mL buffer A. The column was then incubated in 5 mL buffer A containing 50 U of thrombin at 25 °C for 16 h. Protein was eluted from the column by the addition of 10 mL of buffer A followed by 10 mL buffer A containing 250 mM imidazole. Thrombin was removed using a 1 mL HiPrep benzamidine column (GE Healthcare) following the manufacturer’s instructions. The partially purified protein was loaded onto a HiPrep 16/60 Sephacryl S-200 gel filtration column using a Biologic LP system (Bio-Rad) housed within the anaerobic chamber. The column was run at a flow rate of 0.5 ml min^− 1^ with 50 mM Tris pH 8.0, 150 mM NaCl, 10% glycerol, 2 mM DTT. Fractions containing only IscU2, as determined by SDS-PAGE, were pooled, concentrated, and desalted into storage buffer (50 mM Tris pH 8.0, 150 mM NaCl, 10% glycerol) using a PD-10 column. Purified IscU2 was stored under N_2_ at − 80 °C.

For the purification of AcnA all steps were performed anaerobically under an atmosphere of 95% N_2_, 5% H_2_ in an anaerobic chamber (Coy Laboratories). Thawed cells were resuspended in buffer C (20 mM Tris, pH 8.0, 500 mM NaCl) containing a few crystals of DNase I and benzamidine HCl hydrate. Cells were lysed by three passages through a French pressure cell at > 110 MPa. Lysates were centrifuged at 41,000 x *g* and 4 °C for 35 min. The supernatant was passed through a 0.45 μm filter and loaded on a 5 ml Ni^2+^-agarose resin chromatography column pre-equilibrated with 25 ml of buffer C. The column was sequentially washed with 50 mL buffer C, 25 mL buffer C containing 50 mM imidazole, and 25 mL buffer C. The column was then incubated in 5 mL Buffer C containing 50 U of thrombin (Promega) at 25 °C for 16 h. Protein was eluted from the column by the addition of 10 mL of buffer C containing 75 mM imidazole. The partially purified protein was loaded onto a HiPrep 16/60 Sephacryl S-200 gel filtration column and eluted under the same conditions as for IscU2. Fractions containing AcnA, as determined by SDS-PAGE, were pooled, concentrated, and buffer-exchanged into buffer D (20 mM Bis-Tris, pH 6.8). A 1 mL HiTrap Q XL ion exchange column (GE Healthcare) was pre-equilibrated with buffer D and loaded with partially purified AcnA. The column was washed with 20 mL of buffer C containing 200 mM NaCl, then with buffer C containing stepwise-increasing concentrations of NaCl (220–300 mM in 20 mM increments). The flow rate was 1 mL min^− 1^. Fractions containing only AcnA, as determined by SDS-PAGE, were pooled, concentrated, and desalted into storage buffer (50 mM Tris pH 8.0, 150 mM NaCl) using a PD-10 column. Purified AcnA was stored under N_2_ at − 80 °C.

Recombinant IscS2, IscU2, and AcnA were each separately purified at least twice, and similar results were observed with each preparation. Results are included from a single preparation unless noted otherwise. All protein concentrations were determined by the Bradford assay [1] using bovine serum albumin as a standard. Protein purity was analyzed by SDS-PAGE using a 10% gel for AcnA, 12% gel for IscS2, and 15% gel for IscU2. A Broad Range (10–230 kDa) prestained protein ladder (New England Biolabs) was used to approximate the molecular weight of each protein. SDS-PAGE gels were stained with Coomassie Brilliant Blue solution, then destained prior to imaging.

### Reconstitution of IscS2 with PLP

IscS2 (150 μM) was incubated with 3 mM PLP in 50 mM Tris pH 7.2, 150 mM NaCl, at 25 °C for 3 h. Unbound PLP was removed from the IscS2/PLP mix by desalting into storage buffer (50 mM Tris pH 8.0, 150 mM NaCl, 10% glycerol) using a PD-10 column. This sample of IscS2 was designated IscS2^PLP^.

### IscU2 Fe-S cluster reconstitution

Two methods were used to examine IscsU2 Fe-S cluster reconstitution. All steps were performed inside an anaerobic chamber. Chemical reconstitution was carried out during a purification of IscU2. After removal of thrombin, partially pure IscU2 (~ 33 mg of protein) was diluted in 50 mL of 50 mM Tris pH 8.0, 150 mM NaCl, 10% glycerol, followed by the addition of 2 mM β-mercaptoethanol, 138 μM ferrous ammonium sulfate, and 138 μM sodium sulfide. The reaction mix was incubated at 4 °C for 16 h and then concentrated to 2.5 mL using a stirred-cell concentrator (5 kDa MW cutoff). IscU2 was purified from the reaction mix by size-exclusion chromatography as described above and designated IscU2^C-FeS^. IscS2-dependent Fe-S cluster reconstitution of IscU2 was performed by incubating 160 μM IscU2 in 100 mM Tris pH 7.4 containing 8 μM IscS2, 2 mM DTT, 1.6 mM ferrous ammonium sulfate, and 1.6 mM L-cysteine for 1 h at 25 °C. The reaction mix was desalted into storage buffer using a PD-10 column, and aliquots were stored under N_2_ at − 80 °C.

### Determination of the oligomeric state of IscS2 and IscU2

The oligomeric state of IscS2 and IscU2 was determined by size-exclusion chromatography using a HiPrep 16/60 Sephacryl S-200 gel filtration column. The column was run at a flow rate of 0.5 ml min^− 1^ with 50 mM Tris pH 8.0, 150 mM NaCl, 10% glycerol, 2 mM DTT, and calibrated with standard proteins (Low molecular weight standards, Sigma-Aldrich): β-amylase (200 kDa), alcohol dehydrogenase (150 kDa), bovine serum albumin (66 kDa), carbonic anhydrase (29 kDa), and cytochrome *c* (12.4 kDa).

### Cysteine desulfurase assay

Cysteine desulfurase activity was determined by measuring production of sulfide from L-cysteine using the methylene blue method [56]. Assays were performed with 5 μM IscS2 in 50 mM Tris pH 7.5, 1 mM DTT in the presence or absence of 50 μM PLP. Reactions were initiated by the addition of 1 mM L-cysteine in a total reaction of 1 mL and were incubated at 25 °C for 20 min in sealed vials. The reaction was stopped after 20 min by the addition of zinc acetate and sodium hydroxide. The mixtures were developed, and absorbance measured at 670 nm.

Similar assays were performed on *M. acetivorans* WWM73 cell-free lysates. Cell cultures were grown to an OD_600_ between 0.6–0.8, and cells were pelleted in sealed anoxic bottles at 11,000 x *g* for 10 min at 4 °C. Cell pellets were resuspended in 50 mM Tris pH 8.0 containing, 1 mM benzamidine, 1 mM phenylmethylsulfonyl fluoride, transferred to vials, and stored under N_2_ at − 80 °C until use. Cells were lysed by sonication in an anaerobic chamber, centrifuged at 16,000 x *g* for 10 min., and the supernatant saved. L-cysteine desulfurase activity was measured using crude lysate (0.16 to 0.34 mg) as described above. Reaction mixtures were incubated at 37 °C for 45 min before termination by adding 100 μL 20 mM N,N-Dimethyl-p-phenylenediamine dihydrochloride in 7.3 M HCl and 100 μL 30 mM FeCl_3_ in 1.2 M HCl using gas-tight syringes. Color developed over 30 min, then solutions were quickly vented, spun at 16,000 x *g*, and absorbances read at 670 nm.

### Spectroscopy

UV-visible spectra of IscS2 and IscU2 were recorded using a Cary 60 spectrophotometer (Agilent Technologies) housed within an anaerobic chamber. CW EPR spectra were measured at X-band (9 GHz) frequency on a Bruker EMX spectrometer, fitted with the ER-4119-HS high sensitivity perpendicular-mode cavity. The Oxford Instrument ESR 900 flow cryostat in combination with the ITC4 temperature controller was used for measurements in the 4 K to 300 K range using a helium flow. All spectra were recorded with a field modulation frequency of 100 kHz, modulation amplitude of 0.6 mT, and a frequency of 9.386 GHz. Sample-specific conditions are indicated in the figure legends.

### Determination of Fe-S cluster and persulfide content in lysate

Acid-labile and persulfide (sulfane) sulfur concentrations were determined in cell-free lysates from strains WWM73 and DJL60 grown on different sulfur sources using the methylene blue method as above. Cell-free lysate was prepared as described above for L-cysteine desulfurase assays. Soluble protein (0.195 to 0.335 mg) were directly assayed by the methylene blue method (acid-labile sulfur) or were incubated at 37 °C with 1 mM DTT for 60 min in sealed vials prior to sulfide determination to assay reductant-labile (persulfide) sulfur. The persulfide concentration was determined by subtracting the amount of acid-labile sulfur determined in the absence of DTT. Control samples containing DTT without lysate did not produce detectable sulfur.

### Aconitase reconstitution assays

AcnA (56 μM) was rendered to the apo-form by anaerobic incubation on ice with 50 M excess (2.8 mM) EDTA, 20 M excess (1.12 mM) potassium ferricyanide in 50 mM Tris pH 7.2, 150 mM NaCl. After 15 min of incubation, apo-AcnA was desalted using a NAP5 column (GE Healthcare) and stored under N_2_ at − 80 °C in 50 mM Tris pH 7.2, 150 mM NaCl until use.

Apo-AcnA was mixed with a 10-fold molar excess of IscU2^S-FeS^ (or with a 40-fold molar excess of ferrous ammonium sulfate and sodium sulfide, or buffer in control reactions) in 50 mM Tris pH 7.2, 150 mM NaCl, 1 mM DTT and allowed to incubate at room temperature in a sealed anaerobic chamber for up to thirty minutes. Samples of these cluster-transfer (or control) incubations were added to activity assay mixtures to achieve final concentrations of 50 mM Tris pH 8, 0.8 μM AcnA, 20 mM sodium citrate, 250 μM NADP^+^, 1 mM manganese sulfate, and 0.5 units/mL porcine isocitrate dehydrogenase (ICDH). The assay mixtures were immediately read anaerobically for absorbance at 340 nm over 8 min in a spectrophotometer. Samples of AcnA that had incubated with IscU2^S-FeS^ (or controls) for 5 min, 15 min, and 30 min were analyzed.

Aconitase activity was also measured in *M. acetivorans* WWM73 cell lysates, as above. Lysate supernatant was used in place of aconitase. Lysate was divided into aliquots and incubated for 3 hours at room temperature, with some maintained in an anaerobic chamber while another aliquot was exposed to ambient oxygen. After 3 hours, the aerobically exposed sample was quickly made anoxic again by vacuuming and purging with nitrogen. Aliquots of the samples were incubated with IscU2 or IscU2^S-FeS^, or controls, in 50 mM Tris pH 7.2, 150 mM NaCl for 15 min, then aconitase/ICDH assays were performed as above.

### Generation of a *M. acetivorans* iscSU2 deletion mutant

The pseudo-wildtype parent strain, WWM73, and plasmid vectors for genetic manipulation were generously provided by Prof. William Metcalf from the University of Illinois, and are listed in Table S1. The *iscSU2* deletion mutant was generated using pJK301 and methods similar to those previously described [57]. Briefly, homologous regions upstream (US) and downstream (DS) of *iscS2U2* were amplified by PCR, using primers containing *Apa*I and *Hind*III recognition sites for the US region and *Bam*HI, and *Spe*I for the DS region. Each PCR product was digested with the appropriate restriction enzymes and sequentially ligated into similarly digested pJK301. Restriction digestion, ligation, and transformation were all carried out as described above. The complete *iscS2U2* knockout plasmid (pDL214) was confirmed by DNA sequencing (Eurofins). Unless otherwise noted, all procedures described below were performed in an anerobic chamber (Coy Laboratories). *M. acetivorans* strain WWM73 was transformed with approximately 2 μg of pDL214 linearized by digestion with *Not*I, using the liposomal transfection method as previously described [58]. Transformants were selected by spread plating on HS agar plates (0.8% w/v noble agar) containing 125 mM methanol and 2 μg/mL puromycin. The plates were placed in a canning jar along with a vial containing 2 ml of 2.5% sodium sulfide. The jar was sealed and incubated at 35 °C in a standard incubator. Well-isolated colonies were inoculated into HS medium supplemented with 125 mM methanol and 2 μg/mL puromycin. Deletion of *iscS2U2* and replacement with the *pac-hpt* cassette from the pJK301 was confirmed in selected transformants by sequencing PCR products generated with primers listed in Table S1 and genomic DNA isolated from transformants. Once confirmed, the *iscSU2* deletion strain was designated as *M. acetivorans* strain DJL60.

## Supplementary information

**Additional file 1 Table S1.** Strains, plasmids, and primers used in this study. **Fig. S1.** Arrangement of the gene clusters containing *iscS*and *iscU*in *E. coli* and *M. acetivorans* C2A. Other predicted gene functions: MA0806, conserved hypothetical protein; MA0809, DrsEsuperfamily protein; MA0810, SirA-like protein; MA0811, histidine triad protein; MA2714, homoserine O-acetyltransferase; MA2715, O-acetylhomoserine (thiol)-lyase; MA2716, quinolinatesynthetase A; MA2719, helix-turn-helix XRE-family like protein; MA3262, LrgBsuperfamily protein; MA3263, LrgAsuperfamily protein. **Fig. S2.** Amino acid sequence alignment of *M. acetivorans*IscS1–3 with IscSfrom *Escherichia coli* and *Archaeoglobusfulgidus*. The active site residues in *E. coli* IscSare red and boxed. The PLP binding residues in *E. coli* IscSare green. Ec: *E. coli*; Af: *A. fulgidus*. **Fig. S3.** Amino acid sequence alignment of *M. acetivorans*IscU1–3 with IscUfrom *Escherichia coli* and *Archaeoglobusfulgidus*. The Fe-S cluster binding residues in *E. coli* IscUare red, and the conserved aspartate and histidine are green. The HscA-interacting region is boxed. Ec: *E. coli*; Af: *A. fulgidus*. **Fig. S4.** Characterization of recombinant *M. acetivorans* aconitase (AcnA).A) SDS-PAGE analysis of purified recombinant AcnA. B) UV-visible spectrum of purified AcnAafter in vitro reconstitution with iron and sulfur.

## Data Availability

The datasets and/or analyzed during the current study are available from the corresponding author on reasonable request.

## Abbreviations

ICDH: Isocitrate dehydrogenase
AcnA: Aconitase
Fe-S: Iron-sulfur
HS: High-salt
DTT: Dithiothreitol
PLP: Pyridoxal 5′- phosphate
