# Supplementary material for: Methanosarcina acetivorans contains a functional ISC system for iron-sulfur cluster biogenesis
Source: BMC Microbiol. 2020 Oct 23;20:323. doi: 10.1186/s12866-020-02014-z (PMC7585200; doi:10.1186/s12866-020-02014-z)
Supplement: Supplementary file 1 — Additional file 1 Table S1. Strains, plasmids, and primers used in this study. Fig. S1. Arrangement of the gene clusters containing iscSand iscUin E. coli and M. acetivorans C2A. Other predicted gene functions: MA0806, conserved hypothetical protein; MA0809, DrsEsuperfamily protein; MA0810, SirA-like protein; MA0811, histidine triad protein; MA2714, homoserine O-acetyltransferase; MA2715, O-acetylhomoserine (thiol)-lyase; MA2716, quinolinatesynthetase A; MA2719, helix-turn-helix XRE-family like protein; MA3262, LrgBsuperfamily protein; MA3263, LrgAsuperfamily protein. Fig. S2. Amino acid sequence alignment of M. acetivoransIscS1–3 with IscSfrom Escherichia coli and Archaeoglobusfulgidus. The active site residues in E. coli IscSare red and boxed. The PLP binding residues in E. coli IscSare green. Ec: E. coli; Af: A. fulgidus. Fig. S3. Amino acid sequence alignment of M. acetivoransIscU1–3 with IscUfrom Escherichia coli and Archaeoglobusfulgidus. The Fe-S cluster binding residues in E. coli IscUare red, and the conserved aspartate and histidine are green. The HscA-interacting region is boxed. Ec: E. coli; Af: A. fulgidus. Fig. S4. Characterization of recombinant M. acetivorans aconitase (AcnA).A) SDS-PAGE analysis of purified recombinant AcnA. B) UV-visible spectrum of purified AcnAafter in vitro reconstitution with iron and sulfur. [file 12866_2020_2014_MOESM1_ESM.pdf]

Table S1. Strains, plasmids, and primers used in this study

Name	Description	Source or reference
	Strains	
E. coli		
DH5α	Cloning strain	NEB
Rosetta (DE3) pLacI	Recombinant protein expression strain	NEB
M. acetivorans		
WWM73	Δ*hpt*::P*mcrB-tetR*-φC31-*int*-attP	[1]
DJL60	WWM73 Δ*iscSU2*::*pac-hpt*	This study
	Plasmids	
pDL201	pET28a with *iscU2* cloned into *Nde*I & *Xho*I sites	This study
pDL202	pET28a with *iscS2* cloned into *Nde*I & *Xho*I sites	This study
pDL204	pET28a with *acnA* cloned into *Nhe*I & *Xho*I sites	This study
pJK301	Amp^R vector for making markerless deletions in *Methanosarcina* spp.; contains *pac-hpt* flanked by Flp recombinase FRT recognition sites	[2]
pDL214	*iscSU2* knockout plasmid; pJK301 with regions upstream of iscS2 cloned into *Apa*I & *Hind*III sites, downstream of iscU2 cloned into *Bam*HI & *Spe*I sites	This study
	Primers	
IscSNdeF “P2 fwd”	ggtttgCATATGacaattgaaaacagaaccgtttac fwd primer with *Nde*I site to amplify *iscS2*	This study
IscSXhoR “P2 rev”	ggtggtCTCGAGtcaaagagctctgtattcctgaggg rev primer with *Xho*I site to amplify *iscS*2	This study
IscUNdeF “P1 fwd”	ggtggtCATATGgattacagcgctaaggtgttag fwd primer with *Nde*I site to amplify *iscU*2	This study
IscUXhoR “P1 rev”	ggtggtCTCGAGtcagtcccagggctcaagccc rev primer with *Xho*I site to amplify *iscU*2	This study
NheAconF	ggtggtGCTAGCatgagagaaggtctggacccc fwd primer with *Nhe*I site to amplify *acnA*	This study
AconXhoR	ggtggtCTCGAGttatttttcttcaccgaatcccgc rev primer with *Xho*I site to amplify *acnA*	This study
ApausISCF	ggtggtGGGCCCccctgattcccatttgaatcttcc fwd primer with *Apa*I site to amplify *iscSU2* US region for insertion in pJK301	This study
HindusISCR	gttgttAAGCTTcgtagcagagttatccatgtaaacgg rev primer with *Hind*III site to amplify *iscSU2* US region for insertion in pJK301	This study
BamdsISCF	ggtggtGGATCCgggcttgagccctgggactgaatgagcc fwd primer with *Bam*HI site to amplify *iscSU2* DS region for insertion in pJK301	This study
SpedISCR	ggtggtACTAGTgctgttcatgggttgttgaagctggatg	This study

	rev primer with *Spe*I site to amplify *iscSU2* DS region for insertion in pJK301	
Isc2USKOseqF<br>“P3 fwd”	gatgtcaaacgggcgagaaacaagagg<br>fwd genomic primer to confirm *iscSU2* knockout	This study
JK301SupR(new)<br>“P3 rev”	tagtatattacgaatagggcg<br>rev plasmid primer to confirm *iscSU2* knockout	This study
JK301SdownF<br>“P4 fwd”	gctgctggtgaaagagacc<br>fwd plasmid primer to confirm *iscSU2* knockout	This study
Isc2DSKOseqR<br>“P4 rev”	ggaagagttcacggaagaattcccgg<br>rev genomic primer to confirm *iscSU2* knockout	This study

1. Guss, A. M., Rother, M., Zhang, J. K., Kulkarni, G. & Metcalf, W. W. (2008) New methods for tightly regulated gene expression and highly efficient chromosomal integration of cloned genes for *Methanosarcina* species, *Archaea.* **2**, 193-203.
2. Welander, P. V. & Metcalf, W. W. (2008) Mutagenesis of the C1 oxidation pathway in Methanosarcina barkeri: new insights into the Mtr/Mer bypass pathway, *J Bacteriol.* **190**, 1928-36.

Supplemental Figure Legends

Fig. S1. Arrangement of the gene clusters containing *iscS* and *iscU* in *E. coli* and *M. acetivorans* C2A. Other predicted gene functions: MA0806, conserved hypothetical protein; MA0809, DrsE superfamily protein; MA0810, SirA-like protein; MA0811, histidine triad protein; MA2714, homoserine O-acetyltransferase; MA2715, O-acetylhomoserine (thiol)-lyase; MA2716, quinolinate synthetase A; MA2719, helix-turn-helix XRE-family like protein; MA3262, LrgB superfamily protein; MA3263, LrgA superfamily protein.

Fig. S2. Amino acid sequence alignment of *M. acetivorans* IscS1-3 with IscS from *Escherichia coli* and *Archaeoglobus fulgidus*. The active site residues in *E. coli* IscS are red and boxed. The PLP binding residues in *E. coli* IscS are green. Ec: *E. coli*; Af: *A. fulgidus*.

Fig. S3. Amino acid sequence alignment of *M. acetivorans* IscU1-3 with IscU from *Escherichia coli* and *Archaeoglobus fulgidus*. The Fe-S cluster binding residues in *E. coli* IscU are red, and the conserved aspartate and histidine are green. The HscA-interacting region is boxed. Ec: *E. coli*; Af: *A. fulgidus*.

Fig. S4. Characterization of recombinant *M. acetivorans* aconitase (AcnA). A) SDS-PAGE analysis of purified recombinant AcnA. B) UV-visible spectrum of purified AcnA after in vitro reconstitution with iron and sulfur.

FIGURE S1

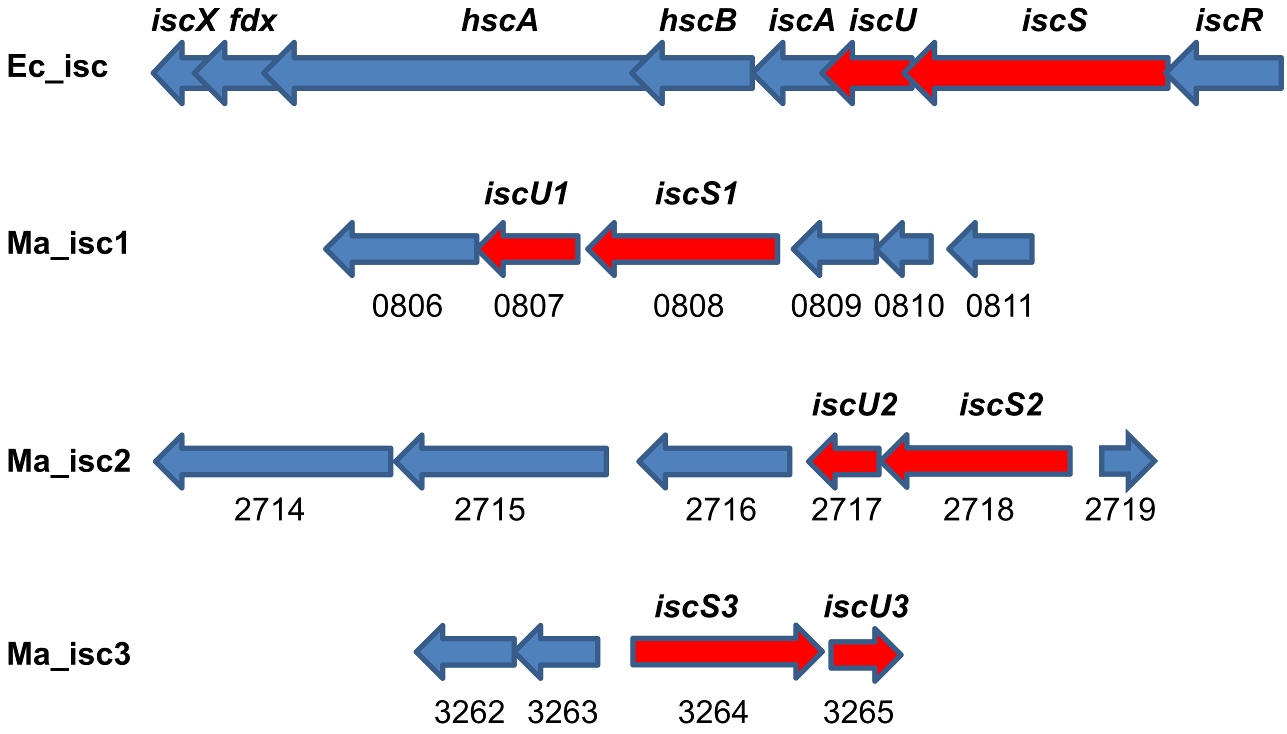
iscX fdx
hscA
hscB
iscA iscU
iscS
iscR
Ec_isc
iscU1
iscS1
Ma_isc1
0806
0807
0808
0809 0810
0811
iscU2
iscS2
Ma_isc2
2714
2715
2716
2717
2718
2719
iscS3
iscU3
Ma_isc3
3262
3263
3264
3265

FIGURE S2

```
IscS1           ---------------------------------------------------------MIYL
IscS2           ---------------------------------------------------MTIENRTVYM
IscS3           MLSSRLFSQDYFPQIHSHLRYFHTFMMQTERFQKAHNKFISSKLLFSLGVLMGETHLIYM
AfIscS          ---------------------------------------------------------MAYF
EcIscS          ------------------------------------------------------MKLPIYL
                                                          *:

IscS1           DNAACTRLDERVFEAMKPYFFDT-YAVATSEFGYSMGIDAKEGLENSREGIASGLGAAP-
IscS2           DNSATTPVRKEVVEEMLPYLTEN--FGNPSS-IYELGKISKHAVENARKRVADAIGAEE-
IscS3           DHAATTFTKPEVIEAMLPFLKEH--FGNPSS-LYSIGREGKEAVETSRKKLAKALGAAQP
AfIscS          DYTSAKPVDERVLEAMLPYMTES--FGNPSS-VHSYGFKAREAVQEAREKVAKLVNGGG-
EcIscS          DYSATTPVDPRVAEKMMQFMTMDGTFGNPASRSHRFGWQAEEAVDIARNQIADLVGADP-
                * :: .    .* * *  ::        .:.  :  *  ....:: :*: :*. :..

IscS1           EEIVFTSGDTESSNMALKGVAWALREKKGKHIIISKIEDFPVLNTAKTLQKQGFDVTFLD
IscS2           NEIYFTSGGTESDNWTVKGVAFAN-KNRGKHIITSSIEHHAVLHACAWLEGQGFEVTYLP
IscS3           EEIYFTSGGTESDNWAIKGTAFSR-QKKGKHIITTPIEHHAVLYPCEYLETQGFEVTYLP
AfIscS          GTVVFTSGATEANNLAIIGYAMRN-ARKGKHILVSAVEHMSVINPAKFLQKQGFEVEYIP
EcIscS          REIVFTSGATESDNLAIKGAANFY-QKKGKHIITSKTEHKAVLDTCRQLEREGFEVTYLA
                  : **** **:.* :: * *     .:****: :  *. .*: ..  *: :**:* ::

IscS1           VDAEGFADLEELKKAITKETILVSIQHSNQEIGTAQDLKAISEICEEKDVLLHTDATHSF
IscS2           VDRYGMVSPEELKNAIRDDTILISIMLANNEIGTIQPVEEIGKISRENSIYFHTDAVQAI
IscS3           VDEYGLVNPAEVEASIKKDTVLISVMYANNEIGTIEPILEIGKIAREHGIPFHTDAVQVI
AfIscS          VGKYGEVDVSFIDQKLRDDTILVSVQHANNEIGTIQPVEEISEVLAG-KAALHIDATASV
EcIscS          PQRNGIIDLKELEAAMRDDTILVSIMHVNNEIGVVQDIAAIGEMCRARGIIYHVDATQSV
                    *  .   :.  : .:*:*:*:   *:***. : :  *.::        * **.  .

IscS1           TRLPLNVKDLP--VDLVTMSAHTIHGPRGIGALCIRKD--TPIVKFMDGGFQEFNLRAGV
IscS2           GHVPIDVKKMN--VDLLSLSGHKFGGPKGCGALYIRKG--TKIEAFLHGGAQERKRRAGT
IscS3           GKVPLDLQREHKDVDMLSLSSHKFYGPKGIGALYIREG--TEIDNYMHGGAQERGKRAGT
AfIscS          GQIEVDVEKIG--ADMLTISSNDIYGPKGVGALWIRKE--AKLQPVILGGGQENGLRSGS
EcIscS          GKLPIDLSQLK--VDLMSFSGHKIYGPKGIGALYVRRKPRVRIEAQMHGGGHERGMRSGT
                 :: :::.     .*::::*.: : **:* *** :*.   . :   : ** :*   *:*

IscS1           ENIPGAVGFATAVKLVTEEENRQLAAMRDR--VIERALSEIPEVTLNGSREKRLPQNANL
IscS2           ENVPSIVGLGKAIGLATGEMEETNKPLLEMRERLIKGLLQIPKTHLNGHPTERLANNVNV
IscS3           ENVVGIVGMGKAIELATANIEAHNEKLSKMRARLMAGILEIPYCRLNGHPEKRLPGNLNF
AfIscS          ENVPSIVGFGKAAEITAMEWREEAERLRRLRDRIIDNVLKIEESYLNGHPEKRLPNNVNV
EcIscS          LPVHQIVGMGEAYRIAKEEMATEMERLRGLRNRLWNGIKDIEEVYLNGDLEHGAPNILNV
                  :   **:. *  :.  :       :      :   : .*    ***   .  .   *.

IscS1           TFHYVEGESVTLHMDMRGFAVSTGSACFSRSLEASHVIRGIGGDHERAHGSVRFTFGRYN
IscS2           TFEYIEGESLLLLLNAKGIFASTGSACNSTSLEPSHVLTACGVPHEIVHGSLRLSLGRMN
IscS3           SFEYIEGESLLLMLDQMGICCSTGSACSSGSSEPSHVLRAIGLAPKTAQGTLRLTLGDAN
AfIscS          RFSYIEGESIVLSLDMAGIQASTGSACSSKTLQPSHVLMACGLKHEEAHGTLLLTLGRYN
EcIscS          SFNYVEGESLIMALKDLAVSS--GSACTSASLEPSYVLRALGLNDELAHSSIRFSLGRFT
                 * *:****: : :.  ..    **** * : :.*:*: . *   : .:.:: :::*  .

IscS1           RMEDADAAIDAMSEIVARLREISPLAKK---------------
IscS2           TLEDVDRVLEVLPEIVQKLRNMSPLTPQEYRAL----------
IscS3           SEEDIDYVLEVLPEVVGKLRAISPFYKPESKCEK---------
AfIscS          TDEDVDRLLEVLPGVIERLRSMSPLYRR---------------
EcIscS          TEEEIDYTIELVRKSIGRLRDLSPLWEMYKQGVDLNSIEWAHH
                  *: *  :: :   : :** :**:
```

FIGURE S3

```
IscU1           MKIEKKSEGIKFPYSEKVLEHFRNPHNVGKIENAD---GKGLEGSPACGDMVAVYIKVEP
IscU2           -----------MDYSAKVLDHFSNPRNMGSIANCD---GVGEVGNAKCGDIMKIFLKVEG
IscU3           ------------MYNKKVMDHFMNPRNVGEIEDAD---GIGEAGNP-HGDQMKIFLKIRD
AfIscU          ------------MYSDKVFDHFQNPRNVGKIEDAD---GVGTVGNPVCGDLMTIYIKVKD
EcIscU          -----------MAYSEKVIDHYENPRNVGSFDNNDENVGSGMVGAPACGDVMKLQIKVND
                             *. **::*: **:*:*.: : *   * *  * .  ** : : :*:.

IscU1           ETKVIEDVKFESYGCASNIATGSVITDLAKGKTLDEAKKITWKQASEELGGLPPIKAHCS
IscU2           D--RIVDVKFQTFGCGAAIASSSMATELIKGKILKEAWELSNKAVAEALDGLPPIKMHCS
IscU3           N--RIEDVKFKTFGCAAAIASSSMATELIKGKTLEEAWELTNEDVAEALEGLPPGKLECS
AfIscU          N--RIEDIKFQTFGCAAAIATSSMATEMAKGKTIEEALKITRDAVAEALGGLPKQKMHCS
EcIscU          EG-IIEDARFKTYGCGSAIASSSLVTEWVKGKSLDEAQAIKNTDIAEELE-LPPVKIHCS
                :   * * :*:::**.: **:.*: *:  *** :.**  :.    :* *  **  * .**

IscU1           VLAVEGLRAAIRDYEEKHGLVTEKEPTTEDVVRRRLKHVMNPLTGLDIIRTNLILKISVE
IscU2           MLAEEAIHEAINDYLQKKG-------------LEPWD-----------------------
IscU3           VVSREATHMAINDYRKKQG-------------LEPLK-----------------------
AfIscU          NLAADALRRAIVDYFRKNGKIDKIKELGLEKELEKMEKGEMDDHGEYCEA----------
EcIscU          ILAEDAIKAAIADYKSKREAK---------------------------------------
                 :: :. : ** **  *.

IscU1           AGVVRVVVDLPEDHQFAPAIKEDIIEKLGALWDVEKVDVVFTA
IscU2           -------------------------------------------
IscU3           -------------------------------------------
AfIscU          -------------------------------------------
EcIscU          -------------------------------------------
```

FIGURE S4

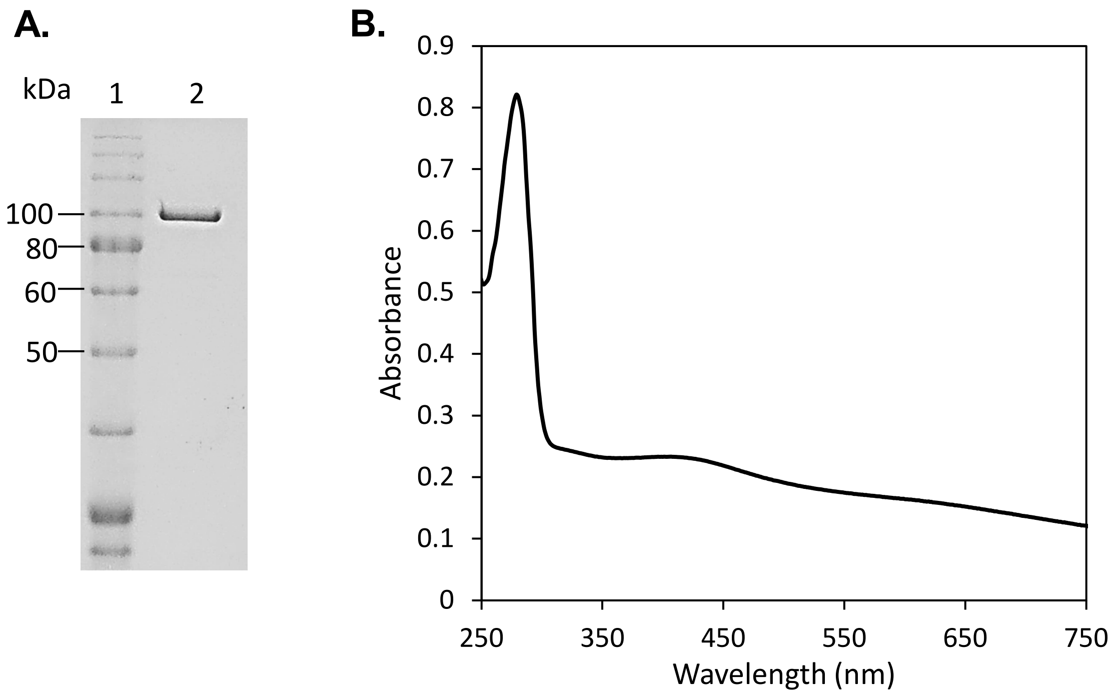
A.
kDa
1
2
100
80
60
50
B.
Absorbance
0.9
0.8
0.7
0.6
0.5
0.4
0.3
0.2
0.1
0
250
350
450
550
650
750
Wavelength (nm)
